# IT development in radiology - an ESR update on the Digital Imaging Adoption Model (DIAM)

**DOI:** 10.1186/s13244-019-0712-z

**Published:** 2019-02-28

**Authors:** European Society of Radiology (ESR)

**Affiliations:** European Society of Radiology (ESR), Am Gestade 1, 1010 Vienna, Austria

**Keywords:** Benchmarking, Information technology, Medical imaging, Strategic planning, Health care evaluation mechanisms

## Abstract

The Digital Imaging Adoption Model (DIAM), a joint project established in 2016 by the European Society of Radiology (ESR) and the Healthcare Information and Management Systems Society (HIMSS), is designed to assist imaging institutions in implementing increasingly integrated IT systems and improving patient care. The model provides a framework through which existing capacities can be assessed and strategy for future institutional development elaborated. DIAM has already been adopted by 58 leading institutions in 18 countries. This article will first provide an overview of the DIAM framework; subsequently, it will consider what its adoption has revealed so far, both through the analysis of global data and through specific case studies; finally, it will outline the future potential and goals of the project.

## Key points

- Introduction to and explanation of DIAM
- Setting out the benefits of DIAM assessment
- Overview of DIAM’s adoption to date
- Looking forward to the expansion of Enterprise Imaging

## Introduction

As radiology was one of the first specialties for which computerization became obligatory for daily work, it is already widely digitized. Support systems such as radiology information systems (RIS) and picture archiving and communication systems (PACS) are standard in most hospitals. However, developments in imaging informatics and information technology have been disrupting the status quo, with the advent of electronic health records (EHR), the Internet of Things, augmented reality, deep learning and cloud-based computing, just to name a few. Adoption of these new technologies in healthcare institutions tends to be slow and piecemeal; therefore, there remains room for significant improvement. DIAM is designed to facilitate the efficient adoption of technology in imaging departments.

## What is the Digital Imaging Adoption Model (DIAM)?

DIAM is the first imaging IT maturity model of its kind worldwide and is aimed at hospitals with imaging departments, external imaging centres that collaborate with hospitals and, potentially, also outpatient-oriented imaging networks [1]. The model exists to support imaging departments in the planning and implementation of imaging IT strategy and to address the challenges related to digitization and improving patient outcomes. DIAM provides thought leadership and guidance with respect to IT-supported processes in medical imaging by helping users and buyers of medical imaging technology to efficiently identify existing, and potential future, infrastructure or workflow gaps and by supporting clinical and managerial users of medical imaging technology in reaching decisions on strategic, operational and procurement levels. This is intended to ensure that departments adopt the appropriate digital strategy, taking account of their circumstances, thus maximising the improvement in health outcomes that can be obtained. Fact-based evidence on the current capabilities of imaging IT is kept under review by ESR and HIMSS experts to guarantee the reliability of DIAM’s results and recommendations. This is based on DIAM’s development as a vendor-neutral tool, supported by a panel of subject matter experts with experience across a broad range of imaging IT fields.

The DIAM framework, with stages from 0 to 7, allows hospitals to gain a clear overview of their existing capabilities; assists in strategic, operational and procurement decisions; and offers the potential for benchmarking with other organizations. If repeated, the DIAM assessment can facilitate the monitoring of progress in imaging IT performance within individual organizations over time. The insights this provides can have both internal and external applications. As in the HIMSS Electronic Medical Records Adoption Model (EMRAM), which has been successfully implemented by over 9000 hospitals worldwide, stage 0 indicates low maturity and stage 7 represents the most advanced maturity of imaging IT. The hope is for DIAM to achieve similar influence and success to EMRAM but within the specific field of radiology and, in the future, through the Enterprise Imaging (EI) initiative, also in other medical imaging domains.

The DIAM model is based on the ten key areas that HIMSS Analytics and the ESR consider to be essential to the digitization of imaging departments:

1. Software infrastructure
2. Health information exchange
3. Workflow and process security
4. Quality and safety management
5. Patient engagement
6. Clinical documentation
7. Clinical decision support
8. Pervasiveness of use
9. Advanced analytics
10. personalized medicine

In turn, these ten focus areas direct attention towards more than 100 indicators integral to the digitization of imaging workflows.

DIAM provides a simple, three-step pathway to enable participating organizations to identify their level of imaging IT capabilities and highlight key areas for improvement based on the essential areas and indicators described above.

**Fig. 1:**
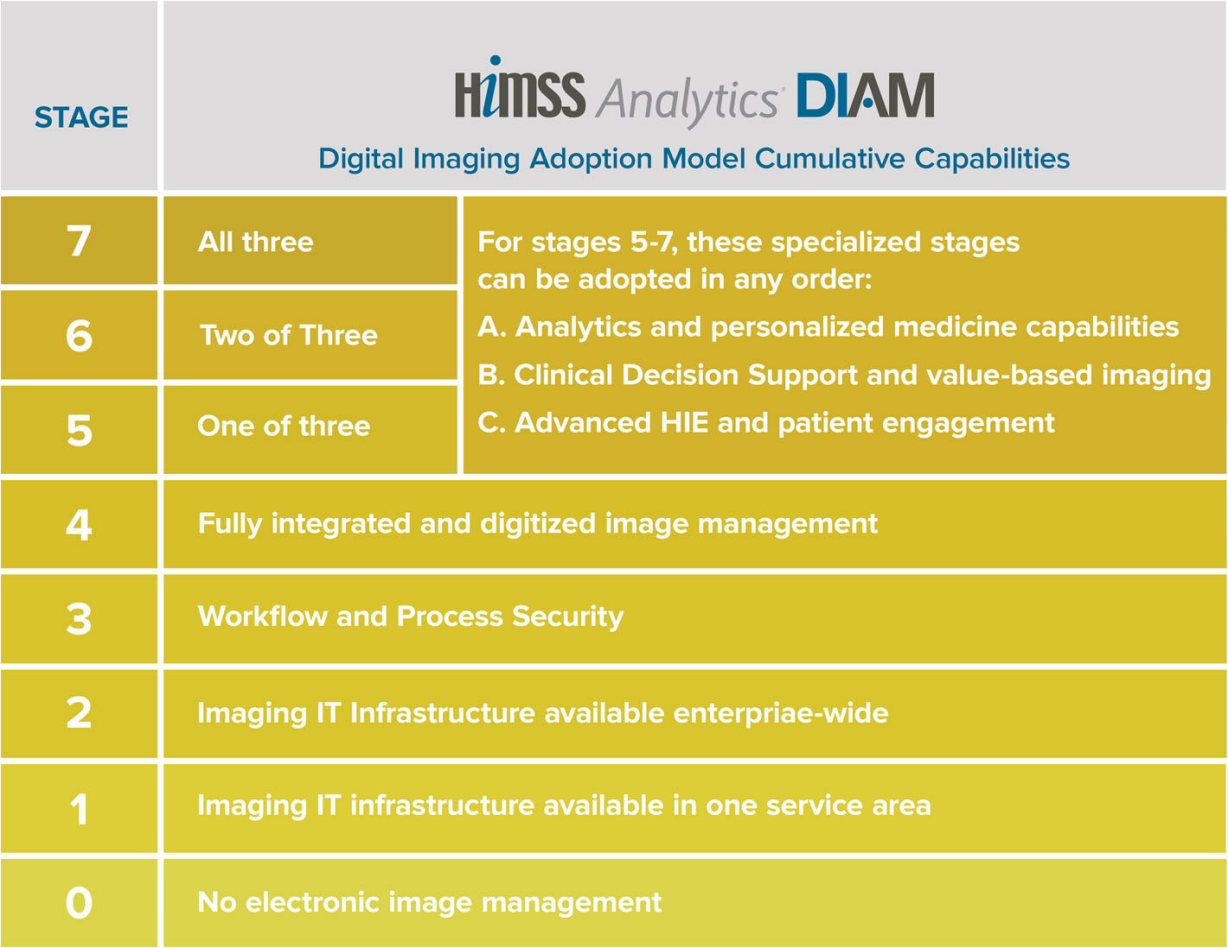
The stages of the DIAM framework

The three-step process consists of the following:

1. An online assessment form, called the DIAM survey, which must be completed by the participating organization. This does not require in-depth IT knowledge; it can be done in approximately 3 hours by radiologists, radiographers, and/or IT-experts, though ideally, it should be a joint effort.
2. A thorough quality assurance assessment, completed by HIMSS after the online DIAM survey is submitted. The extent of this assessment depends on the DIAM level achieved by the submitting organization.
3. A DIAM score and a gap report produced by HIMSS. This score is only shared with the organization which submitted the data. The DIAM score places the participating organization within an 8-stage framework (see Fig. 1).

The gap report can assist in identifying potential improvements in IT infrastructure, workflow and procurement/investment planning. Such information can be of significant value in making sure that departmental strategy is aligned with the overall business strategy of the organization. Additionally, the fact that DIAM is an external assessment makes it valuable in situations in which internal requests or judgments might be more easily overlooked (e.g. negotiations for funding with hospital boards).

## The DIAM framework

**Fig. 2:**
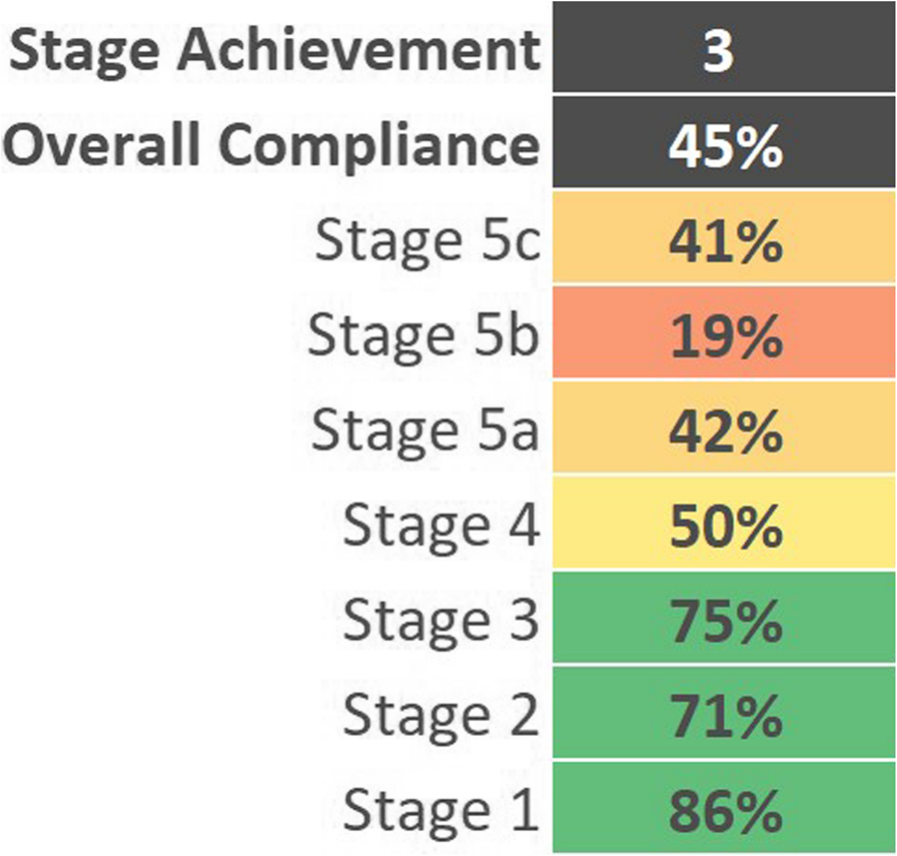
In this example case, the hospital would have achieved DIAM stage 3; although, it has also made some progress towards higher levels too

In stages 0–4, the model has sequential compliance goals, i.e. the organization must meet the requirements of the lower stages before moving to the higher stages. To be awarded a particular stage, at least 70% of its criteria must be met (see Fig. 2). The early stages (0–4^1^) are concerned with the planning and implementation of imaging IT. The following are some (non-exhaustive) examples: To achieve stage 1, the organization should have implemented key software applications relevant for managing the workflows as well as the images and reports acquired and produced in the imaging department, such as a RIS, a PACS or a vendor neutral archive (VNA). In addition, the image management system (IMS) must support the exchange of images and reports with at least 5% of all units of the organization (inpatient and outpatient). To achieve stage 2, these percentages rise to at least 50% and the IMS and RIS should be (at least partially) integrated with the EHR or the hospital information system (HIS). By stage 3, electronically supported processes for matching patient and examination identifiers ought to be in place. At this stage, it is also expected that software tools are used to manage radiation dose and that some degree of clinical decision support (CDS) is implemented, e.g. access to imaging knowledge databases/libraries or alert systems for duplicate imaging referrals. By stage 4, IMS and RIS are expected to be fully integrated with the EHR/HIS as well as being fully capable of seamlessly receiving and processing clinical referrals and images from all patient units, emergency departments and collaborating hospitals. By this stage, secure standards for external data exchange should have been implemented, and exam scheduling and work list management ought to be fully digitized and integrated with EHR/HIS.

Stages 5–7 are non-hierarchical and allow for different approaches when making best use of the most advanced software-related features. If an organization qualifies for stages 6 or 7, an additional validation process will take place before certification. This additional validation process takes place on-site and is to ensure that the DIAM survey has been correctly understood and completed; to confirm that system capabilities, workflows, and processes conform to the DIAM stage 6/7 standards; and to facilitate public recognition for successfully validated organizations so that they can serve as best practice examples. Once the stage is certified, the organization and its achievement are publicised (with the organization’s consent) in media resources, such as on the ESR and HIMSS websites.

As previously noted, the DIAM score is designed to assist in benchmarking and alignment of best practices with other institutions—regionally, within a specific country, or around the world. This benchmarking can help identify organizations that have already made sophisticated and valuable use of imaging technology in order to highlight them as best practice cases at HIMSS and ESR-hosted events. The score can also play a role in discussions and negotiations (e.g. with regional health authorities or IT suppliers); it can provide grounds for access to additional funding, etc.; and it can assist in identifying where new appointments are needed and/or where there is a need for the education and training of existing staff. Furthermore, it assures patients and the public of the organization’s overall quality. As such, the DIAM score, the use of the DIAM logo on an organization’s website and promotion at major HIMSS and ESR events can be significant promotional tools for a participating institution.

The intention is to build a network of peers and facilitate the sharing of smart solutions to common challenges. Although participation has, thus far, been limited, it is hoped that as the DIAM project expands and more hospitals consent to their data being shared, peer networking/referencing can be expedited and that this shall be a valuable resource for DIAM participants. This should create a feedback loop in which the DIAM project becomes more valuable as more organizations join, in turn attracting more organizations.

DIAM is designed to assist in the transition to value-based radiology [2], allowing healthcare providers to maximize their efficiency and optimise patient outcomes. Value, recognized as quality relative to cost, can be promoted through the avoidance of unnecessary procedures or the use of cheaper procedures where any associated reduction in image quality is inconsequential to diagnosis. Thus, DIAM may also be considered a route towards the implementation of value-based imaging, e.g. by encouraging the use of CDS programmes such as ESR iGuide.

DIAM is not only of utility to healthcare providers, but also offers demonstrable benefits to vendors as follows: it offers the advantage of standardization, it provides access to preference knowledge in relation to imaging IT, it can assist in identification of key market opportunities and it can drive business strategy and tactical alignment.

Having explained what the DIAM model is, how it works, and what it is intended to do, this article will now give an overview of conclusions that can be drawn regarding the state of digital imaging based on the data collected from the organizations that have been assessed so far. It shall then focus on short case studies of four specific organizations in different countries and at different DIAM stages to demonstrate the positive impact the DIAM assessment can have in terms of day-to-day practice and patient care.

## DIAM’s implementation and impact

As of August 2018, 58 organizations from 18 different countries in Europe, North America, the Middle East, and the Asia-Pacific region had been surveyed (see Table 1). The majority of these organizations were hospitals (86.2%), followed by other organizations (10.3%) and finally diagnostic imaging centres (3.4%). The mean DIAM score is 3.1,^2^ which demonstrates that significant progress has been made in digitization of imaging workflows and also highlights the potential for further improvements, both in Europe and further afield.

**Table 1:** Countries with the number of their participating institutions

Country	N	Type of Organization
Netherlands	14	13 Hospitals, 1 National Cancer Screening Programme
Germany	8	6 Hospitals, 1 Primary Care Radiology Network, 1 Diagnostic Imaging Centre and Hospital
Russia	8	7 Hospitals, 1 external Diagnostic Imaging Centre
Spain	6	5 Hospitals, 1 Primary Trust and Hospital Association
Italy	3	Hospitals
Sweden	3	Hospitals
USA	3	2 Hospitals, 1 Multi Hospital System
Saudi Arabia	2	Hospitals
United Kingdom	2	Hospitals
Belgium	1	Hospital
Finland	1	Regional Imaging Centre
Ireland	1	Hospital
Kuwait	1	Hospital
Poland	1	Hospital
Singapore	1	Hospital
Switzerland	1	Hospital
Ukraine	1	National Scientific Centre
UAE	1	Hospital
TOTAL	58	

Only one institution has so far achieved a DIAM score of 6 or more (see Table 2). This was achieved in November 2017 by the King Abdulaziz Medical City (KAMC) in Riyadh, Saudi Arabia. Based on the key areas considered essential to the digitization of imaging by the ESR and HIMSS, a general impression of the state of digital imaging adoption rates on a global scale can be provided and various gaps in digital imaging adoption can be identified. Such information may be of value not just to institutions considering participation in DIAM, but to all imaging facilities.

**Table 2:** DIAM scores by institution

Digital Imaging Adoption Model ^SM^		
Stage	Results (August 2018)	
Stage 7	0	0%
3 out of 3 achieved		
Stage 6	1	2%
2 out of 3 achieved		
Stage 5	4	7%
1 out of 3 achieved		
Stage 4	21	36%
Stage 3	8	14%
Stage 2	16	28%
Stage 1	4	7%
Stage 0	4	7%
N	58	

## Key gaps identified by DIAM assessments so far

This overview of different institutions from many different countries is, of course, still based on a relatively small sample size; however, it clearly indicates different topics which should be focused upon. Within the ten key areas that form the basis of the DIAM model, six in particular can be identified as in need of further improvement if digital imaging is to be considered successfully adopted based on analysis of the so-far completed DIAM assessments. These are health information exchange, patient engagement, clinical decision support, pervasiveness of use, advanced analytics and personalized medicine. Additionally, it is possible to identify two further areas that must be considered for the successful adoption of digital imaging: interoperability and structured reporting.

### Health information exchange

The geographic scope of external data exchange activities has been shown to be limited: although 91% of the organizations assessed stated that local exchange is at least partially enabled, at a regional level, this drops to 79%. On a country-wide level, only 7% of organizations can exchange images to the full extent (47% have partial capabilities). At cross-country level, perhaps unsurprisingly, no organization reports to have strong capabilities to exchange images with other organizations; however, a few (19%) indicate having at least some such capabilities.

### Patient engagement

Only 36% of participating organizations make diagnostic images and reports from the imaging department available to patients through an electronic platform such as a patient portal or a similar application. Making appointments for imaging examinations online is possible for patients in less than a quarter of the organizations. Less than a third of organizations offered eConsultation (i.e. specialists give input through email-based e-consults in order to reduce need for patients’ in-house visits), and only 16% provide virtual/online follow-up routine care (e.g. in patients’ home or office).

### Clinical decision support (CDS)

The DIAM assessments completed thus far reveal that less than half of the participating organizations have CDS tools integrated into their electronic workflow. Assuming that organizations without such tools integrated are less likely to have participated in the first place (to avoid receiving a low score, even though the scores are not made public without their consent), this would suggest that there is significant room to expand the adoption of CDS systems.

Of the organizations surveyed, 38% report having access to medical knowledge databases/libraries dedicated to imaging (e.g. annotated imaging studies, diagnosis, patient cases) through the software used in their imaging department. Furthermore, 36% stated the software used in their imaging department automatically provides alerts for duplicate imaging referrals for the same patient.

However, only 5% use software which provides support in identifying the appropriate imaging exam or diagnostic test (depending on patients’ symptoms and using evidence-based guidelines), such as the ESR’s CDS system ‘ESR iGuide’. Integrated directly in the referral workflow, ESR iGuide utilizes the ESR’s imaging referral guidelines to provide guidance to referring physicians.

In addition, 84% fail to use an electronic reminder system to track compliance for appropriate follow-up examinations (e.g. mammogram, abdominal lesions, cysts). This appears to be a great opportunity to improve health and avoid unnecessary hospital admissions in the future that are currently being missed. Such ‘low-hanging fruit’ demonstrates how DIAM can identify opportunities to maximize cost-benefit in planning IT strategy.

Evidence-based recommendations from CDS are only monitored in order to analyse clinicians’ compliance and/or the validity of the recommendations in 11% of the organizations surveyed, though 28% use software which provides hyperlinks between report text (e.g. diagnosis, quantity) and image area.

While computer-aided detection (CADe) to automatically mark conspicuous structures and sections of medical images is used, at least to some extent, in many organizations (60%), computer-aided diagnosis (CADx) is only (at least partially) used to automatically evaluate/pre-interpret conspicuous structures and sections of medical images in 33% of the surveyed organizations. Only 9% use computer-aided simple triage (CAST) for automatic initial interpretation and triage of studies into meaningful categories. The use of detection and diagnosis tools that are based on artificial intelligence and deep learning capabilities is rarely seen, most of the functions mentioned above being based on ‘traditional’ algorithms.

CDS features, like the suggestion of alternative examinations or suggestions for standardised care practices/best-practice guidelines, are rarely directly integrated in electronic workflows (7%, 12%).

DIAM, through its promotion of CDS, has the potential to benefit both hospitals and patients by expediting workflows, by avoiding the need to seek pre-approval of procedures from insurers, and by reducing the incidence of inappropriate procedures being carried out.

### Pervasiveness of use

This focus area is about making sure that organizations make full use of the technologies they have already implemented, consistently and across various users/employees. As an example, if an imaging department has implemented speech recognition software with licences for all staff members, the expectation would typically be that this solution would be used by all radiologists. The organization should thus monitor its radiologists to ensure they actually do use the software in question and, if the data shows that some are using it less often than others, investigate the reasons for this and take appropriate actions to improve the situation (e.g. by offering additional training). Similar experiences were made with the implementation of CDS for imaging referrers, where lack of user adoption can be a barrier to unlocking the full potential of improved imaging utilization. Awareness among users and adequate training and support should be complemented by administrative, financial, clinical or regulatory incentives. During a stage 6/7 validation, the organization being surveyed is expected to provide key statistics to demonstrate to the inspecting team that usage of implemented technologies meets certain targets over a given time period. Evidence from the DIAM surveys so far completed suggests that currently implemented technologies are not always optimally utilized.

### Advanced Analytics

One third of the surveyed facilities lack any formal analytics and/or business intelligence strategy. The use of predictive risk analysis of patients (e.g. to calculate the risk of developing cancer to identify cancer spots in a certain geographical area) remains rare: just 17% of the facilities use departmental or enterprise-wide data, and only 9% use external data (e.g. population data or insurance data).

### Personalized medicine

Personalized medicine [3] is still an area that displays some key gaps: out of five items addressing this in the DIAM survey, four are not used by the majority of the surveyed facilities. The biggest gap is radiogenomics, i.e. correlating genetic information from patients with imaging biomarkers in order to optimise the treatment: 88% of facilities reported that they do not make use of such tools/data. Furthermore, 71% do not use in vivo molecular imaging (i.e. the visualization, characterization, and measurement of biological processes at the molecular and cellular levels), and 72% of the facilities fail to actively facilitate the concept of theranostics, i.e. the integration of personalized diagnostic imaging and therapeutic intervention (in order to improve therapy effectiveness and minimal invasiveness).

Systems to support the use of structured information based on imaging biomarkers for image post-processing and quantification in order to optimise the treatment (e.g. type, length, intensity) are slightly more common, but those facilities reporting that they use them (33%) remain in the minority.

In addition to the above key areas, the DIAM assessments have so far highlighted two additional areas that require further improvement if digitization of imaging is to be fully achieved:

#### Interoperability

While encrypted/secure DICOM connection is used by the majority (77%) of entities for external data exchange, other formats and standards like WADO (web access to DICOM persistent objects) (35%), IHE XDS-I (cross-enterprise document sharing for imaging) (37%) or IHE XCA-I (cross-community access for imaging) (18%) are much less frequently used.

#### Structured reporting

Although many (especially older) radiologists are more comfortable with free-text reports, it is clear that, in general, referring clinicians prefer reports in structured formats [4, 5]. Structured reporting templates validated by joint expert panels of the ESR and Radiological Society of North America (RSNA) are available for incorporation into RIS systems. Nonetheless, structured templates for diagnostic image reports conforming to clinical document architecture (CDA), integrating the healthcare enterprise (IHE), management of radiology report templates (MRRT), or comparable standards are used in less than half of the surveyed entities (39%). Over 77% of those completing the DIAM assessment reported sharing less than 5% of reports captured in structured, discrete, computer-readable format conforming to CDA Level 2 (or similar) or higher standards. Only 13% of the assessed organizations reported sharing more than 95% of reports in such a format.^3^

Users should check their IT strategies to assess if such advanced topics should be included for further decision making, vendors should use this information for product management and providing appropriate solutions, and finally, when sufficient results are available to allow regional or national comparison/benchmarking, decision makers should incorporate this into healthcare politics.

## DIAM experiences from the real world

In addition to the general global conclusions that may be drawn from the DIAM data as to the status of digitalisation in radiology, it is also instructive to enquire about the experience of specific hospitals that have completed the DIAM assessment. For this reason, four case studies have been selected to give brief examples of some of the impacts DIAM has had on institutions with differing levels of digitization.

## The King Abdulaziz Medical Centre (KAMC), Riyadh, Saudi Arabia

KAMC is at the forefront of global imaging IT implementation. On the basis of its unique status as the only organization to have been awarded DIAM stage 6 or higher, KAMC provides a useful case study of DIAM’s benefits. Already an EMRAM stage 6 facility, its next goal is to attain DIAM stage 7 together with achieving EMRAM stage 7. In practical terms, the DIAM assessment gave additional impetus to projects such as the implementation of a VNA, an enterprise-wide Master Patient Index (MPI), cross reporting and analytics, which, in turn, had a positive impact on patient care. As an example, over 50,000 duplicate records were discovered among Ministry of National Guard Health Affairs facilities. Care providers are now seeing single, unified patient imaging history records, which enable them to increase efficiency.

Before the KAMC completed its DIAM assessment, many requests for new features and services were made without valid justification. However, after the DIAM assessment, it was noticed that requests for enhancement are now significantly more likely to be justified and, thus, enable IT value. With this in mind, investment in open application provider interfaces (APIs), implementation of HL7 standards and patient-centric IT enhancement requests were ranked as a first priority.

KAMC is now focusing on filling the gaps to fulfil DIAM stage 7 requirements and has started implementation of a new CDS for imaging referrals.

## Moscow Health Department Hospitals, Russia

The Moscow Health Department was swift to recognize the value of the DIAM project and its hospitals began participating soon after DIAM’s launch in 2016. To date, six hospitals from the Moscow Health Department have participated in the DIAM project, the highest ranked of which achieved stage 3. The main gap that needs to be solved for all the Moscow Health Department’s participating hospitals is the provision of seamless electronic exchange of referrals, images and reports, both enterprise-wide and cross-enterprise.

Workflow automation and informatization require daily decisions about the implementation and use of digital technologies. DIAM has assisted healthcare managers in the Moscow Health Department in developing their digitalisation strategy, evaluating the informatization of imaging departments and identifying IT-related problems/shortcomings and possible solutions. Heads of imaging departments in Moscow hospitals now use DIAM action points to build their own data-driven strategies and to justify software implementation.

The ability to build cooperation between different clinics, between clinics and vendors and between different vendors is highly valued. The Moscow Health Department hopes its hospitals will soon be ready to use DIAM as a basis for moving towards the next stages of imaging IT implementation.

## University Medical Centre Groningen (UMCG), Groningen, The Netherlands

The UMCG was the first centre in the Netherlands to perform a DIAM evaluation, achieving DIAM stage 4. In filling out the DIAM assessment, the need for a real team effort to gather all information and to get a balanced response became clear. The perception of certain IT developments can vary depending on who you ask: for example, an IT manager may perceive a certain IT solution to be advanced and properly installed/implemented, while users might have a different perception of its use in practice. DIAM was particularly useful in highlighting such discrepancies.

On the basis of UMCG’s early experiences, one of the main IT consulting companies in the Netherlands decided to receive training on DIAM and be assessed by HIMSS in order to be able to assist hospitals in using DIAM. Now, DIAM training and workshops are organised in the Netherlands and an active campaign has been started to get as many healthcare institutes as possible to perform the DIAM analysis. This lobby was necessary because the initial small number of participating institutes did not allow easy comparison on a national level. To date, these efforts have convinced 14 hospitals to participate (see Table 1). It is hoped that further efforts will result in sufficient numbers to allow national DIAM benchmarking to be established.

## University Medical Centre (UMC) Mainz, Germany

UMC Mainz was one of the first centres evaluated by DIAM, achieving a score of stage 4. In particular, gaps were identified in decision support, dose management software and patient engagement. For all of these three topics, IT projects have been initiated and aligned with the general IT strategy of UMC; procurement processes are ongoing for all of these topics and UMC is aiming to complete reassessment with the goal of achieving a higher DIAM stage in 2019.

## DIAM looking forward—moving beyond the imaging department

Healthcare providers face many challenges related to image sharing: the integration of multiple imaging sources and formats, the standardization of workflows, the engagement of patients, the use of CDS, the improvement of clinical outcomes, and the demonstration of value. Enterprise viewers are ‘being deployed as part of electronic health record implementations to present the broad spectrum of clinical imaging and multimedia content created in routine medical practice today’ [6].

To keep up with such trends, HIMSS Analytics—in collaboration with professional societies including the ESR, the Society of Imaging Informatics in Medicine (SIIM) and the European Society of Medical Imaging Informatics (EUSOMII), along with highly regarded subject matter experts from across Europe, North America, Asia, the Middle East, and Latin America—has been working towards extending DIAM into the area of EI [7].

As a general concept, EI has been defined as ‘a set of strategies, initiatives and workflows implemented across a healthcare enterprise to consistently and optimally capture, index, manage, store, distribute, view, exchange, and analyse all clinical imaging and multimedia content to enhance the electronic health record’ [8]. Its primary aim is to deliver all forms of imaging to the EHR.

The key objectives of the DIAM EI initiative are to make sure of the following:

- the model comprises the key ingredients needed to successfully and comprehensively implement, deploy and enable EI capabilities;
- the model and the underlying assessment are vendor- and country/healthcare system-agnostic;
- Stage 7, while a challenge, is achievable given the availability of key cutting-edge technologies and related solutions.

EI goes beyond radiology imaging to include ophthalmology, dermatology, cardiology etc., as well as non-diagnostic imaging, such as digital photos to document wound care or endoscopy imaging during surgery. Additional criteria for EI include imaging governance, data privacy and a focus on multi-disciplinary collaboration. Governance should be addressed early in the EI process—initially, individual departments typically maintain and control their own imaging operations, data governance, IT support staff and infrastructure. There is usually little cross-departmental sharing of knowledge or infrastructure, which may even produce negative behaviour as the result of historical ‘turf wars’. Many operational workflows may drive the creation of images, and the traditional model of supporting content based on the medical specialty that performs the imaging is becoming obsolete as, increasingly, ‘any provider with appropriate training and security access may store content in the EHR, sometimes bridging historical specialty lines’ [8]. For these reasons, it is vital to ‘start anew with a core group of constructive stakeholders, executives, and sponsorship in overseeing all imaging activities and service lines’ [8].

Artificial intelligence is also likely to play a bigger and bigger role in clinical imaging. Virtual or augmented reality is also likely to increase in the coming years. Increasing patient engagement (e.g. patients being able to add data to their records) will also potentially have significant impacts.

To help drive these developments, the European Training Curriculum for Radiology published by the ESR has, since 2018, required a more extensive range of imaging informatics knowledge and skills. Also, first steps have been taken in the development of a sub-specialism of imaging informatics radiologists in order to train the next generation of radiologists that are able to advance radiology using imaging informatics tools. Knowledge about DIAM and how to implement it is part of this training curriculum.

The DIAM EI initiative entered its pilot phase in October 2018 and it is planned to be launched before the ECR 2019. The extended DIAM EI model will not make the DIAM model for radiology obsolete/redundant as the EI initiative’s broader applicability reduces its potential for detailed, department-based analysis of weaknesses etc. As such, organizations will be given the choice to select their preferred type of evaluation or to use the two models alongside each other to maximize the depth and breadth of the assessment.

## Conclusion

DIAM is easy to apply and, based on the gap report results, delivers relevant information to participating institutions, which can be used to adapt IT strategies and investment decisions accordingly.

DIAM can act as a guide for radiologists to advocate for necessary developments, as a clear pathway for managers to understand the need for information technology deployment in patient care and as a menu for governments and healthcare systems to inform their allocation of resources with a view to optimizing patient outcomes.

## Abbreviations

APIs: Application provider interfaces
CADe: Computer-aided detection
CADx: Computer-aided diagnosis
CAST: Computer-aided simple triage
CDA: Clinical document architecture
CDS: Clinical decision support
DIAM: Digital Imaging Adoption Model
EHR: Electronic health records
EI: Enterprise imaging
EMRAM: Electronic Medical Records Adoption Model
ESR: European Society of Radiology
EUSOMII: European Society of Medical Imaging Informatics
HIMSS: Healthcare Information and Management Systems Society
HIS: Hospital information system
IHE XCA-I: Cross-community access for imaging
IHE XDS-I: Cross-enterprise document sharing for imaging
IHE: Integrating the healthcare enterprise
IMS: Image management system
KAMC: King Abdulaziz Medical City
MRRT: Management of radiology report templates
PACS: Picture archiving and communication system
RIS: Radiology information system
RSNA: Radiological Society of North America
SIIM: Society of Imaging Informatics in Medicine
UMC: University Medical Centre
UMCG: University Medical Centre Groningen
VNA: Vendor neutral archive
WADO: Web access to DICOM persistent objects

## Footnotes

1 It should be noted that where stage 2 refers to ‘enterprise-wide’ imaging infrastructure, this refers to the ability to access radiological images via one viewer across the enterprise; it is distinct from ‘Enterprise Imaging’, which will be discussed further on.

2 Excluding hospitals that were awarded a ‘stage 0’ rating.

3 These figures have been corrected to account for data reported by three organizations that were not deemed wholly credible (which itself demonstrates the thoroughness of the quality assessment and provides justification for the on-site assessments required for higher DIAM stages). As all figures derived from the DIAM assessments rely on self-reported data, a degree of inaccuracy is inevitable. However, the picture revealed is nonetheless indicative of a clear polarization.
